# An overview of arsenic trioxide-involved combined treatment algorithms for leukemia: basic concepts and clinical implications

**DOI:** 10.1038/s41420-023-01558-z

**Published:** 2023-07-27

**Authors:** Yanan Jiang, Xiuyun Shen, Fengnan Zhi, Zhengchao Wen, Yang Gao, Juan Xu, Baofeng Yang, Yunlong Bai

**Affiliations:** 1 Department of Pharmacology (State-Province Key Laboratories of Biomedicine-Pharmaceutics of China, Key Laboratory of Cardiovascular Research, Ministry of Education), College of Pharmacy, Harbin Medical University, Harbin, China; 2 Translational Medicine Research and Cooperation Center of Northern China, Heilongjiang Academy of Medical Sciences, Harbin, China; 3 Department of Gastrointestinal Medical Oncology, Harbin Medical University Cancer Hospital, Harbin, China; 4 College of Bioinformatics Science and Technology, Harbin Medical University, Harbin, China; 5 Research Unit of Noninfectious Chronic Diseases in Frigid Zone, Chinese Academy of Medical Sciences (2019RU070), Harbin, China

**Keywords:** Cancer therapy, Drug development

## Abstract

Arsenic trioxide is a first-line treatment drug for acute promyelocytic leukemia, which is also effective for other kinds of leukemia. Its side effects, however, limit its clinical application, especially for patients with complex leukemia symptoms. Combination therapy can effectively alleviate these problems. This review summarizes the research progress on the combination of arsenic trioxide with anticancer drugs, vitamin and vitamin analogs, plant products, and other kinds of drugs in the treatment of leukemia. Additionally, the new progress in arsenic trioxide-induced cardiotoxicity was summarized. This review aims to provide new insights for the rational clinical application of arsenic trioxide.

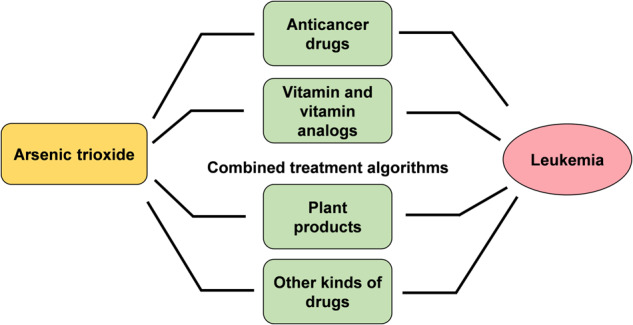

## Facts

- Arsenic trioxide is a first-line treatment drug for acute promyelocytic leukemia in clinic.
- Arsenic trioxide can induce serious side effects, which limit its clinical application.
- There were a lot of drug combination strategies for alleviating arsenic trioxide-induced side effects and improving the curative effects.
- Combination therapy was becoming an achievable strategy for the treatment of leukemia with arsenic trioxide.
- Plant products are more likely to be used in combination with arsenic trioxide.
- Noncoding RNAs are involved in the mechanisms of the arsenic trioxide-induced cardiotoxicity.

## Open questions

- How should we do to find the efficient and pivotal combination therapy for alleviating arsenic trioxide-induced side effects, and improving its curative effects on leukemia?
- What is the key therapeutic mechanism of combined medication?
- What are the advantages of plant products combined with arsenic trioxide?
- What regulatory role does noncoding RNA play in the therapeutic mechanism of arsenic trioxide combination therapy?

## Introduction

Arsenic trioxide (As_2_O_3_) is the main component of traditional Chinese medicine “Pishuang”, which has an application history of over 2000 years. In the 1970s, Zhang Tingdong from Harbin Medical University, China, uncovered the extraordinary therapeutic effect of arsenic trioxide solution on acute promyelocytic leukemia (APL) (The classification of acute leukemia was shown in Table 1). Since then, the anti-leukemia effect of arsenic trioxide monotherapy was extensively explored, achieving highly complete remission rate in both newly diagnosed and relapsed/refractory APL patients [1–3]. In early twenty-first century, arsenic trioxide was approved for the treatment of APL, myelodysplastic syndrome, and multiple myeloma by U.S. Food and Drug Administration (FDA). Nevertheless, therapy with arsenic trioxide may be associated with some side effects, especially cardiotoxicity, which limits its clinical application [4–6]. Besides, arsenic trioxide monotherapy has been shown less effective in non-APL acute myeloid leukemia (AML) (lacking t (15;17) translocation) patients [7–9]. Therefore, it is important to develop new strategies to promote the therapeutic efficiency of arsenic trioxide and to reduce its toxicity. Combination therapy is a frequently used strategy to alleviate this problem. This review summarizes the research progress of arsenic trioxide combined with anticancer drugs, vitamins and vitamin analogs, plant products and other kind of drugs in the treatment of leukemia, in order to provide new insights for the treatment of leukemia and drug combination research.

**Table 1:** The classification of acute leukemia.

FAB classification system		WHO classification system	
**AML**		**AML**	
M0	Acute myeloid leukemia with minimal differentiation	AML with minimal differentiation	Acute myeloid leukemia with minimal differentiation
M1	Acute myeloid leukemia without maturation	AML without maturation	Acute myeloid leukemia without maturation
M2	Acute myeloid leukemia with maturation	AML with maturation	Acute myeloid leukemia with maturation
M3	Acute promyelocytic leukemia	APL	Acute promyelocytic leukemia
M4	Acute myelomonocytic leukemia	AMML	Acute myelomonocytic leukemia
M5	Acute monocytic leukemia		
M5a	Acute monoblastic and monocytic leukemia		Acute monoblastic and monocytic leukemia
M5b	Acute monocytic leukemia		Acute and monocytic leukemia
M6	Erythroleukemia		Acute erythroid leukemia
M6a	Erythroleukemia		Erythroleukemia
M6b	Pure erythroid leukemia		Pure erythroid leukemia
M7	Acute megakaryoblastic leukemia		Acute megakaryoblastic leukemia
**ALL**		**ALL**	
L1	Acute lymphoblastic leukemia (homogenous small cell)	ALL	Acute lymphoblastic leukemia
L2	Acute lymphoblastic leukemia (heterogeneous large cell)	ALL	Acute lymphoblastic leukemia
L3	Acute megakaryoblastic leukemia (homogeneous large cell)		Burkitt-type acute lymphoblastic leukemia

*ALL* acute lymphocytic leukemia, *AML* acute myeloid leukemia, *AMML* acute myelomonocytic leukemia, *APL* acute promyelocytic leukemia, *FAB* French-American-British, *WHO* World Health Organization.

## Arsenic trioxide combined with anticancer drugs

### Cytotoxic anticancer drugs

#### DNA/RNA synthesis inhibitors

Fludarabine, a nucleotide analog of aryladenosine, has a significant effect on chronic lymphocytic leukemia (CLL) [10]. However, drug resistance is still a problem during fludarabine treatment [11]. CLL cells with chromosome 17p13 deletion are predisposes to fludarabine resistance. Whereas, this subtype is more susceptible to arsenic trioxide. Arsenic trioxide at 2 and 4 μM, preferentially induced apoptosis of B-cell CLL cells from patients with unfavorable prognosis (including those that were resistant to fludarabine), compared to B cells from healthy controls [12]. A further study showed that arsenic trioxide (3 μM) facilitated apoptosis of B-cell CLL cells from patients by the suppression of PI3K/Akt signaling pathway [13]. Arsenic trioxide (1 μM) and fludarabine (5 μM) have a synergistically anti-leukemia effect in untreated and fludarabine-resistant CLL cells from patients. This effect was associated with decreased phosphorylation levels of Akt and ERK, as well as decreased Mcl-1/Bim and Bcl-2/Bax ratios [14]. Therefore, the combination of arsenic trioxide and fludarabine may be an effective therapeutic strategy for overcoming fludarabine resistance in CLL patients.

#### DNA methyltransferase inhibitors

Decitabine monotherapy or in combination with chemotherapy was effective in refractory and relapsed AML patients [15]. Decitabine and arsenic trioxide could inhibit proliferation of MV-4-11 AML cells (The summary of leukemia cell lines were shown in Table 2) in a concentration dependent manner, with IC50 values of 2.409 and 2.364 μM (2 × 10^4^ cells each well in 96 well-plate, 48 h), respectively. Decitabine (5 μM) and arsenic trioxide (2 μM) play a synergistic effect on inhibiting the proliferation and inducing apoptosis of MV-4-11 AML cells [16].

**Table 2:** Leukemia cell lines.

Cell name	Organism	Disease
EL4	Mus musculus	Lymphoma
HL-60	Homo sapiens	Acute promyelocytic leukemia
Jurkat	Homo sapiens	Acute T-cell leukemia
K-562	Homo sapiens	Chronic myeloid leukemia
KG-1	Homo sapiens	Acute myelogenous leukemia
KG-1a	Homo sapiens	Acute myelogenous leukemia
MOLM13	Homo sapiens	Acute myeloid leukemia
MOLM14	Homo sapiens	Acute myeloid leukemia
MT-2	Homo sapiens	T-lymphocytic leukemia
MV-4-11	Homo sapiens	Biphenotypic B myelomonocytic leukemia
NALM-20	Homo sapiens	B-cell acute lymphoblastic leukemia
NAMALWA	Homo sapiens	Burkitts lymphoma
NB4	Homo sapiens	Acute promyelocytic leukemia
OCI-AML3	Homo sapiens	Acute myeloid leukemia
SU-DHL-4	Homo sapiens	B-cell lymphoma
SUP-B15	Homo sapiens	Acute lymphoblastic leukemia
THP-1	Homo sapiens	Acute monocytic leukemia
TOM-1	Homo sapiens	Philadelphia chromosome-positive acute lymphoblastic leukemia
U-937	Homo sapiens	Histiocytic lymphoma
WEHI-3	Mus musculus	Leukemia

#### Anti-cancer antibiotics

Idarubicin is an anthracycline anticancer drug that can be used as a monotherapy in the treatment of acute non-lymphocytic leukemia [17]. In a clinical study, eight relapsed APL patients were enrolled to evaluate the therapeutic efficacy of a single induction course with arsenic trioxide (10 mg/day) followed by consolidation therapy with idarubicin. The combination of idarubicin and arsenic trioxide was effective in achieving molecular remission in relapsed APL patients, and was reducing the long-term toxicity of arsenic trioxide and the mutagenicity of combination chemotherapy [18].

In addition, the increased dose of arsenic trioxide (0.5 mg/kg) in combination with idarubicin and cytarabine was safe and well tolerated in newly diagnosed AML patients. Arsenic trioxide supplementation improved the outcomes of patients compared with those who received cytarabine and idarubicin. Therefore, arsenic trioxide may enhance the efficacy of chemotherapy, which may be related to the downregulation of Stat3 [19].

#### Protein synthesis inhibitors

Homoharringtonine is an approved anti-leukemia drug, which mainly exist in the plants of Cephalotaxus Sieb. et Zucc. ex Endl. Homoharringtonine (30 ng/ml) and arsenic trioxide (4 μM) could synergistically promote the apoptosis of U-937 AML cells, which was related to the inhibition of PI3K/Akt signaling pathway and its downstream molecule MCL-1 protein expression [20]. A recent case report indicated that the combination of homoharringtonine and arsenic trioxide bring substantial effects on an elderly patient with AML1/ETO [21]. However, the underlying mechanisms still need to be elucidated.

Paclitaxel, an ingredient from taxus chinensis, is one of the most prominent anticancer drugs. However, paclitaxel was not effective in refractory or relapsed acute lymphocytic leukemia (ALL) patients [22]. A synergistic effect of paclitaxel (5 nM) and arsenic trioxide (1 μM) was observed in inhibiting proliferation and inducing apoptosis of Jurkat T-cell ALL cells, which was related to the increased phosphorylation of Cdk1 and formation of the inhibitory spindle checkpoint complex BubR1/Cdc20. In addition, arsenic trioxide also increased the sensitivity to paclitaxel of primary cells from ALL patients [23].

### Noncytotoxic anticancer drugs

#### Tyrosine kinase inhibitors

Tyrosine kinase inhibitors (TKIs) are a common kind of antitumor drugs, which can be divided into multi-targeted and single-targeted TKIs. Clinically, patients with Fms-related tyrosine kinase 3 internal tandem duplication (FLT3-ITD) mutation are the largest subgroup of AML, which is associated with an increased relapse risk and decreased disease-free survival [24].

Multi-targeted TKI sorafenib has favorable initial outcomes in AML patients, but then is limited due to subsequent drug resistance [25, 26]. Patients with FLT3-ITD mutation are the largest group of AML (~25% of all AML patients) with the poorest prognosis [27]. Arsenic trioxide has relative selective activity on FLT3-ITD cells (MOLM14, MV-4-11, and HB11;19) compared with non-FLT3-ITD leukemia cells (HL-60, SEM-K2, THP-1, U-937, and K-562) [28]. When sorafenib (25 nM) combined with arsenic trioxide (1 μM), a synergistic apoptosis inducing effect was observed in FLT3-ITD MOLM13 AML cells, which is related to the inactivation of FLT3-ITD (both expression and phosphorylation) and GSK3β, the upregulation of Bax and Bak and the downregulation of Mcl-1. Furthermore, the combined treatment also prolonged the survival of mice xenografted with MOLM13 cells [29]. Similarly, the combination of arsenic trioxide (1 and 2 μM) and sorafenib/quizartinib (4 and 8 nM) showed synergistic anti-leukemia effects in FLT3-ITD AML cells (MOLM14 and MV-4-11) and primary cells from patients [28]. In addition, arsenic trioxide and sorafenib combination significantly reduced the viability and promoted the apoptosis of non-FLT3-ITD U-937 cells (arsenic trioxide 1 μM and sorafenib 5 μM) and KG-1 cells (arsenic trioxide 2 μM and sorafenib 7 μM), with an increased transcription of pro-apoptotic and autophagic genes [30]. Therefore, the combination of arsenic trioxide and multi-targeted TKIs (sorafenib and quizartinib) has the potential to improve the outcomes of FLT3-ITD AML patients, which may be also effective in non-FLT3-ITD patients.

Single-target TKIs include imatinib, dasatinib, gefitinib, etc. Imatinib (STI571, a BCR-ABL TKI) is effective in the treatment of chronic myeloid leukemia (CML). Du et al. found that the combination of arsenic trioxide (1 μM) and imatinib (0.25 μM) was more effective and efficient than imatinib monotherapy in inducing apoptosis in K-562 CML cells. They also identified a series of altered genes using microarray analysis, which requires further validation [31]. Nilotinib (a BCR-ABL TKI) (5 nM) and arsenic trioxide (1 μM) in combination inhibited the proliferation but promoted the differentiation of CML cells derived from patients with blast crisis [32]. Arsenic trioxide combined with dasatinib (Src and BCR-ABL inhibitor) synergistically inhibited the proliferation and induced apoptosis of Philadelphia chromosome-positive (Ph+) ALL cells (SUP-B15) and negative ALL cells (TOM-1). This effect was through activating the IRE1/JNK/PUMA axis [33]. Gefitinib (a selective EGFR TKI) (10 μM) could enhance arsenic trioxide (0.6 μM) induced differentiation of NB4 APL cells through promoting ROS production and activation of ERK pathway [34]. The Src family kinase inhibitor PP2 (10 μM) significantly promoted arsenic trioxide (0.5 μM)-induced differentiation of NB4 cells through increasing ICAM-1 and cathepsin D expression [35]. And the triple combination of PP2 (10 μM), ATRA (1 nM) and arsenic trioxide (0.5 μM) enhanced the differentiation of APL cells through increasing ICAM-1 expression [36]. Therefore, the combination of arsenic trioxide and TKIs may be beneficial in the treatment of CML, APL and non-APL AML, especially FLT3-ITD AML.

#### Protease inhibitors

Bortezomib is a selective and reversible inhibitor of the 26S subunit of the proteasome. Arsenic trioxide (1 μM) and bortezomib (5 nM) synergistically inhibited the proliferation and induced the apoptosis of HL-60 cells through enhancing caspase activation, mitochondrial depolarization, ROS production and abrogating DNA-binding activity of NF-κB. Through microarray analysis, the pathways related to this drug combination (arsenic trioxide 2.2 μM and bortezomib 5.6 nM) were identified, including “proliferation of leukocytes”, “tumorigenesis”, “control of cell cycle”, “hypoxia”, “oxidative stress”, etc. Moreover, in three cases of AML-M2, arsenic trioxide (2.2 μM) and bortezomib (5.6 nM) showed synergistic anti-leukemia activity [37].

AT7519 is a pan-cyclin-dependent kinase inhibitor, which could reduce cyclins expression and induce G1 cell cycle arrest, thereby inhibiting the proliferation of AML KG-1 cells. The combination of AT7519 (0.75 and 1 μM) and arsenic trioxide (2 µM) resulted in a superior cytotoxicity than either drug alone in inhibiting KG-1 cell growth [38]. However, further investigations are still needed.

#### All-trans retinoic acid

All-trans retinoic acid (ATRA), a metabolic derivative of vitamin A in animals, is efficient in the treatment of APL with a complete remission rate of 90–95% in patients [39]. Unfortunately, relapses frequently accompanied by ATRA resistance, which occur in 15–30% of APL patients [40].

A series of clinical investigations proved the effect of ATRA and arsenic trioxide combined treatment for different condition of patients with leukemia. ATRA combined with arsenic trioxide as first-line treatment can effectively improve the clinical outcomes of newly and relapsed APL [41–45]. Especially, this drug regimen has high efficiency and safety in pediatric patients with APL [46–48]. In addition, a recent case report discovered that arsenic trioxide combined with ATRA was effective and safe for a child with down syndrome and APL, who is often intolerable to cytotoxic chemotherapy [49]. This drug combination regime has also proved to be safe and effective for elderly APL patients [50].

Nucleophosmin-1 (NPM1) gene mutation occurs in about 50–60% of AML patients with normal karyotype [51]. The combination of ATRA and conventional chemotherapy selectively improved the survival of AML patients with NPM1 mutation in the absence of FLT3-ITD [52].

In arsenic trioxide (1 μM) and ATRA (1 μM) synergistically play inhibitive effect on OCI-AML3 and primary AML cells harboring an NPM1 mutation, with little effect on non-APL wild type NPM1 AML cells. The combined effect was through inducing proteasomal degradation of mutant NPM1, restored NPM1 nucleolar localization, PML and SUMO-1 nuclear body formation [53]. These findings indicate that combined therapy can further improve the long-term efficacy and safety outcomes of patients.

Studies have revealed the mechanisms of the combined therapy with ATRA and arsenic trioxide. In vitro studies found that ATRA could increase the uptake of arsenic trioxide by leukemia cells through upregulating a transmembrane protein aquaporin-9 (AQP9), which is recognized as a major pathway of arsenic uptake. The expression of AQP9 was reverse correlated with arsenic trioxide sensitivity in leukemia cells. NB4 cells were sensitive, whereas Jurkat, NALM-20, HL-60, and K-562 cells were relatively resistant to arsenic trioxide treatment [54]. The transfection of AQP9 promoted the sensitivity of K-562 cells to arsenic trioxide [54]. ATRA (100 nM) treatment up-regulated AQP9 expression, resulting in increased arsenic uptake in the HL-60 cells [54]. In addition, curcumin could enhance the uptake of arsenic trioxide in NB4 cells by increasing AQP9 expression, which may improve the therapeutic efficiency of arsenic trioxide [55]. These findings suggest that the increasing of AQP9 expression may be a potential strategy to promote the efficiency of arsenic trioxide. Sumi et al. found that co-treatment with arsenic trioxide (0.5 μM) augmented ATRA (0.1 μM) induced HL-60 cell differentiation, which was through the downregulation of PRTN3 expression, and accompanied by a concomitant increase in Sp1 and IL-1β [56]. These findings provide new insights in explaining the synergism between ATRA and arsenic trioxide.

With the development of monoclonal antibody therapeutics, a triple combination with gemtuzumab ozogamycin was developed. Gemtuzumab ozogamycin, an anti-CD33 monoclonal antibody, is the world’s first antibody drug conjugate to be marketed for leukemia. The combination of ATRA and arsenic trioxide, with or without gemtuzumab ozogamycin, is an effective regimen in patients with newly diagnosed APL [57]. Similar results were achieved by a multicenter phase III study [58]. A long-term data confirmed this chemotherapy-free triple combination strategy, which is effective and safe [59]. Besides, a recent study reported that a patient who relapsed from ATRA and arsenic trioxide was successfully treated with gemtuzumab ozogamycin followed by unrelated cord blood transplantation [60].

Subsequently, other triple-drug therapeutic regimens were put forwarded. Danthala et al. modified the drug dosage and proposed an arsenic trioxide, ATRA, and daunorubicin involved strategy for high-risk APL, which achieved durable responses with minimal toxicity [61]. de Almeida et al. found that the combination of gefitinib could mitigate ATRA and arsenic trioxide resistance of APL cells [62]. Devadas et al. proposed a sequential schedule with arsenic trioxide followed by ATRA and daunorubicin, which showed low toxicity, low mortality, and high cure rate [63]. The combination of arsenic trioxide, ATRA, and anthracycline was safe and effective for newly diagnosed APL regardless of FLT3-ITD mutation status [64, 65]. This triple combination regime was also effective for pediatric patients with APL [66].

In addition, tamibarotene (a synthetic retinoid) is from the same family as ATRA, but is more potent and chemically stable than ATRA. Tamibarotene is active in newly diagnosed or relapsed and refractory APL patients, even after chemotherapy, ATRA and/or arsenic trioxide treatment [67, 68]. Clinically, arsenic trioxide combined with tamibarotene achieved a molecular complete remission in a patient with refractory APL, who was insensitive to arsenic trioxide and tamibarotene monotherapy [69]. Therefore, the combination of arsenic trioxide and tamibarotene may be effective and tolerable for refractory APL cases which have no treatment options.

## Arsenic trioxide combined with vitamin and vitamin analogs

### Vitamin C

Vitamin C, also known as ascorbic acid, is a polyhydroxyl compound that acts as an effective antioxidant. The sensitivity of leukemia cells to arsenic trioxide correlates with intracellular glutathione (GSH) levels. Cells with lower levels of GSH are more sensitive to arsenic trioxide [70, 71]. The arsenic-resistant of cells could be overcome by GSH depletion [72]. Ascorbic acid may improve the efficiency of arsenic troxide by reducing intracellular GSH levels. Ascorbic acid (62.5 μM) combined with arsenic trioxide (1 μM) exert a stronger pro-apoptosis effect compared to single-drug therapy in the HL-60 and SU-DHL-4 AML cells. Whereas, ascorbic acid did not enhance the inhibitory effect of arsenic trioxide on colony formation of normal hematopoietic cells [73]. Vineetha et al. also validated that ascorbic acid can enhance the pro-apoptosis effect of arsenic trioxide on HL-60 cells [74]. In accordance with these findings, Bachleitner-Hofmann et al. found that arsenic trioxide and ascorbic acid have a synergistic effect for patients with AML [75]. While, another clinical study proposed that the combined therapy has limited benefit effect on patients with non-APL AML (relapsed or refractory AML) [76].

### Vitamin D

Paricalcitol (19-Nor-1,25(OH)_2_D_2_) is a noncalcified vitamin D analog. Studies have found that paricalcitol can effectively inhibit the activity of leukemia cells in bone marrow. Paricalcitol could inhibit the proliferation of HL-60, NB4, and THP-1 leukemia cells [77]. Paricalcitol (0.1 μM for NB4 cells and 0.01 μM for HL-60 cells) combined with arsenic trioxide (0.6 μM for NB4 cells and 0.8 μM for HL-60 cells) inhibited the growth and promoted the differentiation and apoptosis of myeloid leukemia cells. This effect may be due to the decreased expression of PML-RARA fusion protein and the vitamin D metabolizing protein by arsenic trioxide, and thus increased paricalcitol activity [78]. 1,25(OH)2D3 is an activate of vitamin D3. The combination of 1,25(OH)2D3 (50, 100 and 200 nM) could promote arsenic trioxide-induced cell cycle arrest and apoptosis. This combined effect was associated with the activation of p21 and p27, as well as the enhanced expression of vitamin D receptor [79].

### Vitamin E

Vitamin E includes eight natural forms. Among them, α-tocopherol is the most abundant and effective one. (+)-α tocopherol succinate (α-TOS), a succinate ester of (+)-α tocopherol, can induce apoptosis of NB4, NB4-R2 (ATRA-resistant) and primary APL cells. A synergistic pro-apoptosis effect was observed in the treatment of NB4 cells when α-TOS (37.68, 75.36 and 150.72 μM) was administered 24 h after arsenic trioxide (1, 2 and 4 μM). On the contrary, coadministration of α-TOS exerted a moderate antagonistic effect on apoptosis induced by arsenic trioxide [80]. For HL-60 cells, coadministration of α-TOS can enhance the pro-apoptosis effect of arsenic trioxide [74]. The strategies that arsenic trioxide combined with vitamin and vitamin analogs still need further investigation, which may improve the clinical outcomes of APL patients with co-morbidities or contraindications to anthracyclines.

## Arsenic trioxide combined with plant products

### Parthenolide

Parthenolide, an active ingredient from Chrysanthemum morifolium, is a NF-κB inhibitor. It has been suggested that selective NF-κB inhibitors may be usefully in increasing the therapeutic effect of arsenic trioxide [81]. Parthenolide (10 µM) increased cytotoxicity of arsenic trioxide (2.5, 5 and 10 µM) in murine and human leukemia cell lines of myeloid and lymphoid origin, including EL4, Jurkat, K-562, HL-60 cell lines [82]. Similarly, the combination of these two drugs (arsenic trioxide 2 µM and parthenolide 1 µg/ml) decreased the viability and increased G1 phase arrest in MT2 adult T-cell leukemia cells, which was related to the downregulation of CD44, NF-κB, BMI-1 and c-MYC [83]. Besides, the addition of buthionine sulfoximine (an γ-glutamylcysteine synthetase inhibitor) further potentiated the effect of the combined treatment. The effect of the triple drug combination (arsenic trioxide 2 µM, parthenolide 1 µM and buthionine sulfoximine 12.5 µM) was related to the decrease of GSH and ATP levels, and the promotion of oxidative stress in leukemia cell lines. Importantly, healthy donor lymphocytes were largely unaffected by the same treatment regimen [82].

### Resveratrol

Resveratrol is a nonflavonoid polyphenol compound. Coadministration of resveratrol substantially amplifies arsenic trioxide inducing autophagy in NB4 cells. The effect of flavonoid genistein was also observed in this project, but less effective than resveratrol [84]. Similarly, another research provided evidence that resveratrol enhances the inhibitory effects of arsenic trioxide on primary AML or CML primitive leukemia progenitors in vitro. Resveratrol (10 and 25 µM) enhanced the pro-apoptotic effect of arsenic trioxide (1 and 2 µM) in KT1 cells, with increased cleaved PARP expression. Low dose of arsenic trioxide (0.5 µM) combined with the same dose of resveratrol could inhibit colony formation in K-562, U-937, KT1 cells and primary cells from CML patients [85]. Further studies proved the combined effect of resveratrol and arsenic trioxide on drug resistant cells. Adriamycin-resistant K-562 leukemia (K-562/RA) cells were cross-resistant to multiple agents (pirarubicin, daunorubicin, 5-FU, etoposide, vincristine and paclitaxel), with the exception of arsenic trioxide. Resveratrol (20 µM) enhanced the proliferation inhibition and apoptosis of K-562/RA and K-562 cells induced by arsenic trioxide (2 µM), which was related to the suppression of drug resistance related genes (P-gp, MRP1 and BCRP) and altered apoptosis-related gene expression [86]. In addition, resveratrol could also alleviates arsenic trioxide-induced cardiotoxicity, hepatotoxicity and nephrotoxicity [87–89]. These findings indicate that resveratrol can synergistically enhance the sensitivity of leukemia cells and alleviate the toxicity of arsenic trioxide.

### Salvianolic acid A

Salvianolic acid A is an antioxidant exacted from the roots of Salviae miltiorrhizae, which have anticancer and cardioprotective efficiency [90, 91]. The combination of Salvianolic acid A (25 µM) and arsenic trioxide (5 µM) have synergistic activity in K-562 and HL-60 cells [92]. Moreover, Salvianolic acid A could attenuates arsenic trioxide-induced injury in H9c2 cardiomyocytes thorough upregulating Bcl-2 expression and downregulating Bax and Caspase-3 protein expression [93]. Similarly, Salvianolic acid A pretreatment was identified to alleviate arsenic trioxide-induced cardiotoxicity both in vivo and in vitro by improving the damaged mitochondrial function, and the maintenance of normal mitochondrial biogenesis [92]. These findings indicate that the Salvianolic acid A could enhance the anticancer activity of arsenic trioxide and alleviate its cardiotoxicity.

### β-Elemene derivative

β-Elemene is an active component of herb medicine Curcuma wenyujin Y.H Chen & C.Ling. It’s derivative N-(β-Elemene-13-yl) tryptophan methyl ester (ETME) (25 µM) alone or in combination with arsenic trioxide (1 µM for NB4 cells and 2 µM for HL-60 cells) can synergistically induces apoptosis in leukemia cells through enhanced production of H_2_O_2_, increased expression of cleaved PARP and Caspase-3. Therefore, ETME combined with arsenic trioxide can synergistically induce apoptosis of leukemia cells [94]. These findings indicate that this drug combination may be useful in leukemia patients who do not responsive to arsenic trioxide alone.

### Hedyotis diffusa Willd extract

Hedyotis diffusa Willd (HDW) is an annual herb that belongs to the Rubiaceae family. HDW extract can promote the immune response and exhibit inhibitory activity in WEHI-3 leukemia BALB/c mice [95]. A further study validated that HDW alone or combined with arsenic trioxide could promote the total survival rate of BALB/c mice bearing WEHI-3 cells and the inhibitory effect on WEHI-3 cells in a dose-dependent manner. This effect was through enhanced expression of death receptor 4, death receptor 5, Bak and Bid, the activation of Caspase-3, Caspase-8 and Caspase-9, and the inhibition of Bcl-2, Bcl-xl and survivin expression [96]. These studies provide preclinical evidence for the potential efficacy of a combined therapy using HDW with arsenic trioxide in the treatment of APL.

### Auraptene

Auraptene is a natural coumarin with anticancer activity [97]. The combination of auraptene and arsenic trioxide synergistically inhibits the viability and induces G1 phase arrest of MT-2 cells through the downregulation of NF-κB (REL-A), CD44, c-MYC, and BMI-1. Therefore, the combined use of auraptene and arsenic trioxide could be considered as a nonchemotherapy regimen for T-cell leukemia [98].

## Arsenic trioxide combined with other kinds of drugs

### Thalidomide

Thalidomide is a glutamic acid derivative with VEGF inhibitive effect. Mohammadi Kian et al. found that thalidomide combined with arsenic trioxide could synergistically inhibit proliferation, promote apoptosis and autophagy of U-937 (arsenic trioxide 1 µM and thalidomide 60 µM) and KG-1 (arsenic trioxide 1.618 μM and thalidomide 80 μM) AML cells. The effect of combination therapy was better than monotherapy. And the molecular mechanism of combination therapy was related to the downregulation of ULK1 and BECLIN1, and upregulation of PTEN and IL6 [99].

### Sulindac

Non-steroidal anti-inflammatory drugs (NSAIDs) have been proven to possess anticancer potential [100]. NSAIDs sulindac sulfide and diclofenac could induce apoptosis and differentiation in THP-1 and HL-60 AML cell lines and primary AML cells from patients [101]. Stepnik et al. found that the combination with sulindac enhanced the cytotoxicity of arsenic trioxide on Jurkat, HL-60, K-562, HPB-ALL and EL4 leukemia cell lines. The combination of arsenic trioxide (1 μM) with sulindac sulfide or sulindac sulfone at concentrations over 50 μM synergistically promoted apoptosis of these cell lines, with little effect on the viability of normal human lymphocytes [102]. Their follow-up study further proved that the cytotoxicity of arsenic trioxide (0.5 or 1 μM) was enhanced by sulindac (100 μM for Jurkat and HL-60 cells, 200 μM for K-562 cells). Interestingly, the metabolites of sulindac (sulindac sulfide and sulindac sulfone) showed higher activity than sulindac. The most effective combination appears to be arsenic trioxide and sulindac sulfide. This combined effect may be due to the induction of apoptosis, but may be not through the induction of ROS production or MAPK pathway activation [103]. Therefore, clinically achievable concentrations of NSAIDs may serve as adjuvant drug for leukemia. However, the combined mechanism still needs to be elucidated.

### Buthionine sulfoximine

Buthionine sulfoximine is effective in depleting the cellular level of GSH [72]. Arsenic trioxide (1 μM) and buthionine sulfoximine (10 μM) synergistically induced apoptosis in NB4, U-937, NAMALWA, and Jurkat cells primarily through the activation of c-Jun NH2-terminal kinase (JNK) and upregulation of death receptor 5 and cleaved Caspase-8. Besides, the degradation of IκBα was only observed by combined treatment but not with either agent alone [104]. In addition, buthionine sulfoximine (50 μM) also promoted the susceptibility of HL-60 cells to arsenic trioxide (3 μM). The combined treatment induces mitochondrial injury and apoptosis in HL-60 cells by promoting the phosphorylation of BIM_EL_ and MCL1. Furthermore, the knockdown of BIM_EL_ abolished the effect of the combined treatment [105].

### Deferoxamine

Deferoxamine, a hydroxamic acid complexing agent, is an anti-leukemia agent, but its effective dose is relatively high. Therefore, studies to lower the effective dose of deferoxamine were conducted. Yu et al. found deferoxamine combined with arsenic trioxide has synergistic inhibitory effect than monotherapy on HL-60 cells in a nude mouse model, with no significant side effects. The combined effect was associated with the upregulation of Caspase-3 and Bax, and the downregulation of NF-κB p65 and survivin [106].

### Rapamycin

The FRAP/mTOR inhibitor rapamycin (also known as sirolimus) is a macrolide immunosuppressive drug. Altman et al. reported that rapamycin (10 nM) enhances the inhibitive effect of arsenic trioxide (1 μM) on primary leukemia progenitors from AML patients [107]. Subsequently, Dembitz et al. found that rapamycin (20 nM) combined with arsenic trioxide (0.5 and 1 μM) has a synergistic antiproliferative and pro-apoptosis effect on AML cell lines (HL-60 and U-937) and primary AML cells that lack typical t (15;17) translocation. This effect may be due to the inhibition of Akt signaling pathway and decrease the expression of anti-apoptotic Mcl-1 protein [108]. The two studies proved the synergistic effect and mechanism of the two drugs at therapeutically achievable doses on non-APL AML cells.

### Mannitol

The combination of arsenic trioxide and ATRA achieved a highly curable rate in APL [94]. Nevertheless, extramedullary relapse, commonly in central nervous system, occurs in 3–5% of APL patients [109]. However, intravenous infusion arsenic trioxide was difficult to reach therapeutic level in cerebrospinal fluid [110]. To bridge this gap, a study found that mannitol infusion could increase permeability of blood-brain barrier for arsenic (AsIII), which was beneficial to central nervous system relapsed APL patients [111]. These findings provide useful insights for optimizing treatment outcomes of arsenic trioxide in central nervous system relapsed APL patients.

### Venetoclax

Venetoclax (ABT-199) is selective Bcl-2 inhibitor proved by FDA for the treatment of AML. The application of venetoclax showed clinical beneficially for newly diagnosed AML [112]. Venetoclax (200 nM) and arsenic trioxide (3 μM) exert a synergistic effect on inhibiting the viability and promoting apoptosis of KG-1 and KG-1a cells. The combined effect was though inhibiting Akt and ERK, leading to GSK-3β activation and Mcl-1 destabilization [113].

### Schiff base oxovanadium complex

Mirjalili et al. found that the IC50 of arsenic trioxide and Schiff base oxovanadium complex on HL-60 cells was 8.37 ± 0.36 μM and 34.12 ± 1.52 μg/ml. Coadministration of extremely low dose arsenic trioxide (0.001 μM) and Schiff base oxovanadium complex (40 μg/ml) could significantly inhibit the cell viability and promote the apoptosis of HL-60 cells, accompanied by the enhanced expression of p21 and p53. Therefore, the combination of these two drugs in the treatment of APL may be with higher efficiency then monotherapy [114].

## Oral-arsenic preparation

### Oral arsenic trioxide

In 1998, researchers at the University of Hong Kong revived oral arsenic or the “modern” liquor arsenicalis to treat APL patients [115]. The research group from the University of Hong Kong and Queen Mary Hospital did a series of works to observe the clinical effect of oral arsenic trioxide [116]. They proposed that oral arsenic trioxide had a short-term efficacy and safety profile similar to intravenous arsenic trioxide [117]. They then found that oral arsenic trioxide, particularly in prolonged maintenance with and ATRA may obviate the need of stem cells transplantation in relapsed pediatric APL patients [118]. Their recent findings proposed a triple combination regimen with oral arsenic trioxide, ATRA, and ascorbic acid maintenance, which was safe and resulted in favorable long-term survival in APL patients. They are still testing this strategy prospectively to further rigorously assess its long-term effects [119]. Recently, Gill et al. reported the results of a clinical study of newly-diagnosed APL from 1991 to 2021. They found that oral arsenic trioxide-based regimens significantly improved all survivals of APL patients. Therefore, arsenic trioxide (intravenous or oral) should be incorporated into all phases of treatment. In addition, the use of an entirely nonchemotherapy in elderly patients should be explored to reduce drug toxicity [120].

### Oral arsenic realgar-Indigo naturalis formula

Another oral arsenic compound, Realgar-Indigo naturalis formula (RIF) with the chemical formula of As4S4, has been shown the highly curative effect for APL treatment, including the adult, pediatric and elderly APL patients.

The researchers from Peking University People’s Hospital did a series of works in comparing the effect of RIF and intravenous arsenic trioxide. They designed a randomized, multicenter, phase III non-inferiority trial to compare the effect of RIF and intravenous arsenic trioxide. They proposed that oral RIF plus ATRA is not inferior to intravenous arsenic trioxide plus ATRA as first-line treatment of APL and may be considered as a routine treatment option for APL patients [121]. They then proposed that oral RIF plus ATRA significantly reduced the medical costs and the length of hospital stay during induction and remission therapy compared with arsenic trioxide plus ATRA in APL patients [122]. A non-inferiority, randomized phase III trial study further validated that RIF plus ATRA is comparable to intravenous arsenic trioxide plus ATRA for non-high-risk APL patients [123]. A subsequent long-term follow-up study further confirmed the effect of this regimen as front-line therapy for non-high-risk APL, and showed that PML-RARA transcript level was associated with relapse [124]. Therefore, this RIF-based chemotherapy-free regimen may be an alternative for non-high-risk APL patients. In addition, they found that RIF and arsenic trioxide showed the similar effects on the recovery of coagulopathy in APL patients [125]. Zhang et al. also validated that RIF application reduces the total hospitalization days and medical costs [126]. This regimen was also reported to be benefit for high-risk APL patients as consolidation therapy [127]. Besides, Li et al. found that sequential application of ATRA, RIF, and chemotherapy shows better efficacy and less toxicity, especially for high-risk patients [128].

Some studies focused on the effect of RIF on pediatric and elderly APL patients. Yang et al. conducted a randomized, multicenter and non-inferiority trial to determine whether intravenous arsenic trioxide can be substituted by oral RIF in the treatment of pediatric APL. They found that RIF is as effective and safe as intravenous arsenic trioxide for the treatment of pediatric APL, with the advantage of reducing hospital stay [129]. Similarly, Luo et al. found that oral RIF can be used as an alternative to intravenous arsenic trioxide for the treatment of pediatric APL patients [130]. Using population pharmacokinetic analysis, Zhang et al. indicated that the AS_4_S_4_ formula is safe in newly diagnosed pediatric APL patients [131]. Liao et al. compared the arsenic concentration in plasma and urinary using RIF and intravenous arsenic trioxide, and found that urine arsenic level can estimate plasma arsenic concentration [132]. Lou et al. validated that RIF plus ATRA may be considered as frontline therapy in newly diagnosed APL patients, especially in elderly patients [133]. There is a case report of using RIF and ATRA in a 92-year-old APL patient [50]. Hang et al. also achieved similar results and proposed that the active ingredients in RIF may target ACHE, NCOA2 and RXRA proteins [134]. In addition, Xie et al. investigated the effect of a novel RIF on NB4 cells, and found that it was more effective than RIF in inducing apoptosis of NB4 cells [135].

These findings suggested that oral arsenic-based regimen is safe and effective, and has a substantial reduction in the duration of hospitalization, which helps to improve the patient’s quality of life. In the further, the appropriate chemotherapy-free regimen for high-risk patients and its long-term efficacy is a challenge in the new era.

## New approach of arsenic trioxide induced cardiotoxicity

Numerous clinical studies reported that exposure to arsenic trioxide, even at a therapeutic dose, may evoke severe cardiac side effects and even sudden cardiac death in some cases [136–139]. Therefore, prophylactic measures used to manage the consequent cardiotoxicity in clinical applications of arsenic trioxide are urgently required. It was widely confirmed that arsenic trioxide-induced oxidative stress, apoptosis, and ion homeostasis imbalance are the important causes of cardiotoxicity [87, 140, 141].

The research group in Harbin Medical University is a pioneer of noncoding RNA-involved arsenic trioxide toxicity research. Their previous study revealed the effect of miRNAs in arsenic trioxide-induced cardiotoxicity for the first time. Arsenic trioxide could upregulate miR-1 and miR-133 expression to induce cardiac electrical remodeling in guinea pig myocardium. The knockdown of miR-1 and miR-133 abolished the cardiac electrical disorders caused by arsenic trioxide through upregulation of Kir2.1 and ERG, respectively [142]. Arsenic trioxide could also upregulate miR-21 and miR-23a expression in hERG-HEK293 cells and neonatal rat cardiomyocytes. And the inhibition of miR-21 and miR-23a expression alleviated arsenic trioxide-induced hERG expression deficiency by targeting Sp1 and Hsp90, respectively [143]. We further found that lncRNA Kcnq1ot1 was involved in arsenic trioxide-induced QT interval prolongation [144]. Subsequently, arsenic trioxide was also reported to inhibit lncRNA NEAT1 expression in H9c2 cardiomyocytes, and the overexpression of lncRNA NEAT1 could attenuated the inflammatory response-induced by arsenic trioxide through inhibiting miR-124/NF-κB signaling pathway [145].

The recent study identified differentially expressed mRNAs, miRNAs, lncRNAs and circRNAs in arsenic trioxide-treated mice myocardium, and provided a comprehensive analysis of differentially expressed genes [146]. Noncoding RNAs are potential targets in elimination arsenic trioxide-induced cardiotoxicity [147]. Still, more researches are needed to further validate the exact mechanisms of arsenic trioxide induced cardiotoxicity.

## Conclusions

**Fig. 1:**
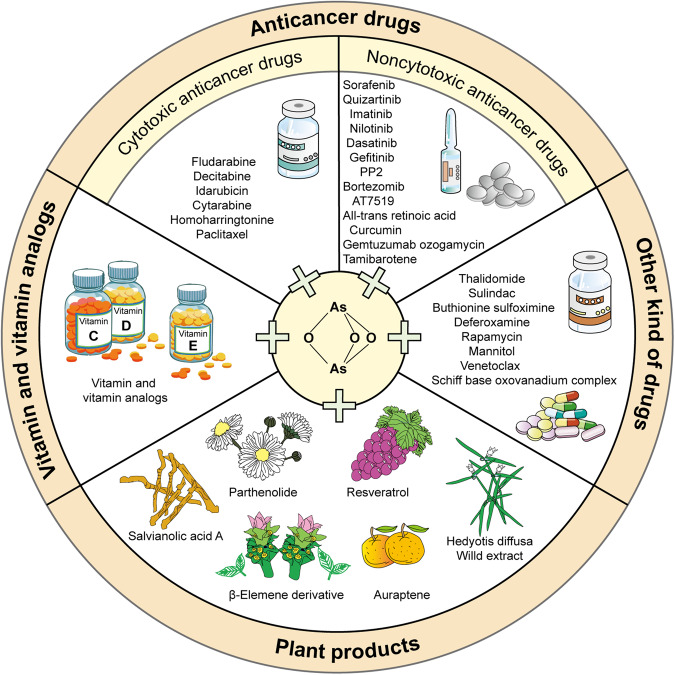
The combined drugs with arsenic trioxide in the treatment of leukemia. The figure summarizes the research progress of arsenic trioxide combined with anticancer drugs, vitamins and vitamin analogs, plant products and other kind of drugs in the treatment of leukemia.

Arsenic trioxide combined therapy is becoming an achievable strategy for the treatment of leukemia (Fig. 1). At present, the combination of arsenic trioxide and anticancer drugs have been used in the treatment of refractory and relapsed leukemia. Other kinds of adjuvant drugs for leukemia are also identified to improve the efficiency of arsenic trioxide, making it available for non-sensitive leukemias. In addition, plant products are more likely to synergistically promote the efficiency of arsenic trioxide and to attenuate its toxicity. However, the mechanisms of combined therapies have not been fully elucidated, which limited the clinical application of these drugs. Noncoding RNAs are expected to become a new mechanism for the arsenic trioxide combined therapy. The present review summarized the current understanding about arsenic trioxide involved drug combination for leukemia (Table 3), in order to provide new insights for the rational use of arsenic trioxide and mechanism study of arsenic trioxide combined treatment for leukemia.

**Table 3:** Combined drugs with arsenic trioxide in the treatment of leukemia.

Combined drug	Leukemia cell type/patient	Clinical report	References
(+)-α tocopherol succinate	NB4 and HL-60 cells	–	[74, 80]
1,25(OH)2D3	K-562 cells	–	[79]
All-trans retinoic acid	HL-60, OCI-AML3, and primary AML cells; APL and AML patients	Yes	[41–45, 49, 50, 53, 54, 56–61, 63–66, 68]
Ascorbic acid	HL-60 and SU-DHL-4 cells; AML patients	Yes	[73–76]
AT7519	KG-1 cells	–	[38]
Auraptene	MT-2 cells	–	[98]
Bortezomib	HL-60 cells; AML patients	Yes	[37]
Buthionine sulfoximine	NB4, U-937, NAMALWA, Jurkat, and HL-60 cells	–	[82, 104, 105]
Curcumin	NB4 cells	–	[55]
Dasatinib	SUP-B15 and TOM-1 cells	–	[33]
Decitabine	MV-4-11 cells	–	[16]
Deferoxamine	HL-60 cells	–	[106]
ETME	HL-60 and NB4 cells	–	[94]
Fludarabine	Primary B-CLL cells	–	[14]
Gefitinib	NB4 cells	–	[34, 62]
Gemtuzumab ozogamycin	APL patients	Yes	[60]
HDW extract	WEHI-3 cells	–	[95, 96]
Homoharringtonine	U-937 cells; AML patients	Yes	[20, 21]
Idarubicin	APL patients, AML patients	Yes	[18, 19]
Imatinib	K-562 cells	–	[31]
Mannitol	APL patients	Yes	[111]
Nilotinib	Primary CML cells	–	[32]
Paclitaxel	Jurkat cells and primary ALL cells	–	[23]
Paricalcitol	HL-60, NB4, and U-937 cells	–	[78]
Parthenolide	EL4, Jurkat, K-562, HL-60, and MT-2 cells	–	[81–83]
PP2	NB4 cells	–	[35, 36]
Rapamycin	HL-60, U-937 and primary AML cells	–	[107, 108]
Resveratrol	NB4, KT1, K-562, U-937, primary AML and CML cells	–	[84–86]
Salvianolic acid A	K-562 and HL-60 cells	–	[92]
Schiff base oxovanadium complex	HL-60 cells	–	[114]
Sorafenib	MOLM13, MOLM14, MV-4-11, U-937, KG-1, and primary AML cells	–	[28–30],
Sulindac	HL-60, K-562, HPB-ALL, EL4, and Jurkat cells	–	[102, 103]
Thalidomide	U-937 and KG-1 cells	–	[99]
Venetoclax	KG-1 and KG-1a cells	–	[113]

*AML* acute myeloid leukemia, *ALL* acute lymphocytic leukemia, *APL* acute promyelocytic leukemia, *CML* chronic myeloid leukemia, *CLL* chronic lymphocytic leukemia, *ETME* N-(β-Elemene-13-yl) tryptophan methyl ester, *HDW* Hedyotis diffusa Willd.
